# Is iron deficiency a risk factor for postpartum depression? A case–control study in the Gaza Strip, Palestine

**DOI:** 10.1017/S1368980021003761

**Published:** 2022-06

**Authors:** Samar Hameed, Ihab A Naser, Mohamed A Al Ghussein, Mohammed S Ellulu

**Affiliations:** 1 Master Program in Clinical Nutrition, Faculty of Pharmacy, Al-Azhar University, Gaza, Palestine; 2 Clinical Nutrition Department, Faculty of Applied Medical Sciences, Al-Azhar University, Gaza, Palestine

**Keywords:** Postpartum depression, Depression, Nutrition, Micronutrient, Trace elements, Iron

## Abstract

**Objective:**

This study aims to investigate the association between iron body status and postpartum depression (PPD) among mothers during the postpartum period.

**Design:**

This is a case–control study.

**Setting:**

Governmental primary health care centres in the Gaza Strip, Palestine.

**Participants:**

This study involved 300 mothers a month after delivery, with one 150 mothers that were recruited in the cases group who were diagnosed with PPD based on Edinburgh Postnatal Depression Scale (EPDS) ≥ 10. The control group included 150 mothers did not have PPD (EPDS < 10). Body iron status is represented by the index of sTfR/log ferritin.

**Results:**

Among PPD mothers, 43·3 % had low ferritin level *v*. 15·3 % for controls (*P* < 0·001) and cases *v*. controls difference in mean Hb level was −0·61 (95 % CI −0·86, −0·35). The results of the multiple logistic regression reported that there is a statistically significant association between PPD and the body iron status existed, as mothers who suffered from iron deficiency (ID) were three times more likely to have PPD (OR^adj^ 3·25; *P* = 0·015). Furthermore, the results of the final regression model showed that the other factors that can lead to PPD are absence of psychological guidance services (OR^adj^ 8·54; *P* = 0·001), suffering from undesired feeling in the last pregnancy (OR^adj^ 1·77; *P* = 0·034), in addition to having one of the mental health disorders in the last pregnancy (*P* = 0·001).

**Conclusion:**

Body iron status might be a risk factor for postpartum depression and other possibilities of reverse causality may worsen the condition.

The WHO considered iron deficiency (ID) as one of the most common forms of nutritional deficiencies, especially among females affecting 33 % of non-pregnant women, 40 % of pregnant women and 42 % of children worldwide^(1)^. ID during pregnancy can cause widespread and significant consequences for both the mother and her infant’s physiological and mental health^(2)^.

Iron has several vital functions in the body including the functions of the iron-containing enzymes, the synthesis of bile acid and steroid hormones, and signal -controlling in some neurotransmitters, such as the serotonin and dopamine systems in brain^(3)^. ID has adverse effects on areas of women’s mental health such as mood, short-term memory, verbal learning, attention, concentration and intelligence. It can also lead to depression^(4)^.

Various physiological variables related to ID that contribute to postpartum depression (PPD) such as fatigue that have been considered as significant predictor of PPD^(5)^. The next variable is anaemia which contributes to fatigue and is associated with additional symptoms such as irritability, apathy and an inability to concentrate^(6)^. Furthermore, ID has been found to have a role in altering thyroid hormone metabolism^(7)^. All of these symptoms (fatigue, anaemia and abnormal thyroid status) are probably interrelated in certain individuals; if they occur during the postpartum period, they could affect maternal psychological and health outcome.

PPD is a major public health concern that may lead to the most common complications of childbearing and represents a serious health problem affecting mothers and their families, and thus the whole society, as it affects about 10–15 % of new mothers^(8)^. However, depression in women during the postpartum period may start during pregnancy or may have onset beyond the first postpartum month^(9)^. The exact aetiology of PPD is still unclear; however, it is generally thought to be linked to genetic, biological, hormonal, psychosocial, environmental and nutritional factors^(10)^.

Generally, balanced nutrition plays an important role in the model of thinking and behaviour, and affects the cognition and memory capacity, and has direct or indirect effects on brain health; as the intake of different foods is directly involved in the synthesis and metabolism of related neurotransmitters^(11)^.

During pregnancy and postpartum periods, mothers face continuous nutritional vulnerability, since malabsorption, food choice, food preparation and other socio-economic factors collectively may have a reverse effect on the micronutrients level including iron^(12)^. Moreover, due to the physiological changes during pregnancy, ID often occurs towards the end of pregnancy even among women who enter pregnancy with some iron stores, which extends to the postpartum period that is associated with the development of maternal PPD^(13)^.

Generally, there are discrepancies in the literature concerning the association between iron and PPD. The direct relationship between ID biomarkers and PPD has been confirmed in different studies^(14–16)^. This relation stems from the fact that iron is critical for adequate myelination, neurotransmitter metabolism and function, and neuronal cellular and oxidative processes^(17)^. In contrast, a study established in China found that there is no relationship between maternal iron status and PPD^(18)^.

This study was conducted in Gaza City, Palestine, due to some perturbing factors. Gaza had been insecure since 2009, due to the dramatic series of events in the region^(19)^. In addition to the socio-economic situation and food insecurity, the Palestinians are exposed to political violence that makes them at heightened risk for major depression and post-traumatic stress disorder. This makes depression one of the top five causes of disability in the occupied Palestinian territory^(20)^. It leads to low socio-economic status resulting in micronutrients deficiencies. This study aims to investigate the association between iron body status and PPD among mothers during the postpartum period in Gaza city.

## Methods

### Study design and sampling

The study population was obtained from the immunisation electronic records of the newly registered neonates in postnatal care. The study involved postpartum mothers, a month (30 d) after delivery, who visited the randomly selected (five out of ten) governmental primary health care centres with their infants for Polio vaccines.

A case*–*control study was conducted in the period from January 2020 to August 2020 in which 1100 postpartum mothers were screened for PPD by using a validated Edinburgh Postnatal Depression Scale (EPDS). The cases were postpartum mothers with depression who scored EPDS (≥ 10), while mothers who scored EPDS (< 10) were not diagnosed with PPD disorder and considered as controls. The controls were paired with the cases according to age and place of residency. Participants who were taking psychiatric drugs during the pregnancy period were excluded from the study. The additional exclusion criteria included mothers who had a history of postpartum blood transfusion, had a postpartum hemorrhage, smokers – as smoking may alter the Hb ratio and increase the oxidative stress – and mothers who refused to sign the consent form.

The study involved 300 mothers, as the sample size was calculated using Power Statistics program for sample size calculation (PS) and by using two-proportion method in Power Statistics software. P0 is the possibility of exposure in the control group = 23·3 % based on the prevalence of ID among non-PPD mothers according to Albacar and his colleagues, while P1 = 38·8 % indicating that 38·8 of the PPD had ID^(14)^. The calculated sample size according to PS software was 144 in each group. After considering the non-response rate, the number of the subjects in each group was increased to 150.

### Questionnaire

A face-to-face interview was conducted to complete a questionnaire designed to meet the needs of the case and control groups. During the interview, the researcher clarified questions for participants. Most questions were closed-ended questions. The questionnaire is divided into three parts: the first part deals with socio-demographic and socio-economic characteristics, the second part contains obstetric and gynecological history, the third part includes the biochemical section.

### Assessment of PPD

The translated copy of EPDS used in this study was established by the Department of Health Government of Western Australia. The cut-off point, recommended by the author, was 9/10, which means a score of 10 or higher indicates that depressive symptoms have been reported. The validation study of this translated questionnaire on Arabic mothers showed that the sensitivity and specificity for the selected cut-off point 9/10 were 91 % and 84 %, respectively^(21)^. Thus, the mothers who scored EPDS (≥ 10) were diagnosed with PPD disorder, while mothers who scored EPDS (<10) were not diagnosed with PPD disorder.

### Blood samples and biochemical measurements

The blood sample collection from the mothers occurred at the newborn’s vaccination section in the selected clinics. The samples were drawn carefully to avoid haemolysis. A 5-ml venous blood sample was drawn from each participant. About 1 and a 1/2 ml were discharged in EDTA vacutainer tubes and mixed gently for the complete blood count test (CBC). The rest of the blood was discharged into the vacutainer plain tube for the biochemical tests and was left to clot then centrifuged at a speed of 3000 rpm for 10 min under room temperature. Then the serum was placed in plain tubes, and in order to avoid loss of bioactivity and contamination, the tubes were sealed and stored at −20°C until the test performed. The biochemical tests that were done from the serum sample are ferritin, hs-CRP and soluble transferrin receptors (sTfR). Each device was calibrated, and controls were performed before each run of samples. All the blood tests were conducted at the Palestinian Medical Relief Society (PMRS) laboratory by the researcher herself.

### Assessment of body iron

In this study, instead of performing the serum iron test, body iron stores were calculated. According to the report of a joint WHO and CDC on the assessment of iron status at the population level, the following equation was used to represent the body iron stores^(22)^:

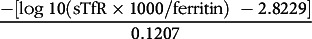




According to Cogswell and her colleagues, after applying the previous equation, the estimate of the prevalence of ID defined as body iron < 0 mg/kg; moreover, they compared these estimates with ID based on the ferritin model among females aged between 12 and 49 years. This means that body iron is expressed as iron excess in stores if the result of sTfR/log ferritin index was ≥ 0 mg/kg. However, if the result was <0 mg/kg, then it indicates iron deficit in the body^(23)^.

Assessment of serum transferrin receptor levels has been used to distinguish ID anaemia from anaemia of chronic disease because the receptors are generally unaffected by concurrent infection or inflammation^(24)^. Combining the use of serum transferrin receptor concentrations with serum ferritin concentrations as the serum transferrin receptor/log ferritin ratio has also been proposed to increase the diagnostic sensitivity and specificity for diagnosing ID^(25,26)^.

### Statistical analysis

Following data collection, the questionnaires and anthropometric data were reviewed before being entered into the study database. Keyed data were checked for missing or unclear responses following completion of the interview. Respondents were contacted by phone to inquire about unclear and missing data.

**Fig. 1:**
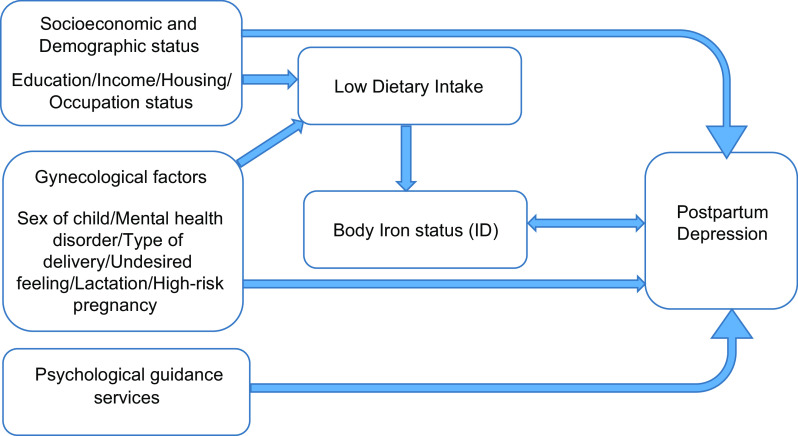
Conceptual frame of the study

Categorical variables like the socio-demographic and obstetric variables were presented as percentages and frequencies, while the quantitative continuous variables, like the biochemical results, were presented as means and standard deviations. The *χ* ^2^ test was used to compare the distribution of categorical qualitative variables across the PPD and non-PPD groups. Independent *t*-test and Mann*–*Whitney test were used to compare between means and ranks, respectively. Multiple logistic regression was performed to investigate the association between body iron status and PPD in present of other confounding factors as shown in Fig. 1. The level of significance was set at 0·05.

## Results

The 300 mothers who participated in this study were categorised into 2 groups: the case group that included 150 mothers who were diagnosed with PPD by the EPDS (≥ 10) with a mean score of 15·2 and sd (± 4·2) and the control group included 150 mothers who were not diagnosed with PPD by the EPDS (<10) with mean score of 5·26 and sd (± 2·65).

Table 1 shows that 85 % of cases and 89·3 % of controls were homeowners and the rest of both groups home renters, thus the type of housing (*P* = 0·856) was not significantly different. Neither the educational level nor the occupational status of the mother showed any significant different as *P*-values were 1·00 and 0·289, respectively. Regarding the total family monthly income for the participant mothers, there was no significant difference between cases and controls (*P* = 0·388). The results showed no statistically significant associations between the PPD and the sex of the child (*P* = 0·298). There were no significant differences in proportions between the two groups regarding the worries about the embryo gender (*P* = 1·000), having the first baby (*P* = 0·250) and planning for the pregnancy (*P* = 0·468). As for the type of delivery, most of the cases and controls had a normal delivery 78·7 % and 76·7 %, respectively, while 21·3 % of cases and 23·3 % of controls had caesarean section (C.S.) delivery, which indicates the absence of the significant differences between PPD and the type of delivery (*P* = 0·782). Despite that the mothers in the control group were breast-feeding 60·0 % compared to the cases group 50·7 %, the results in this study, no statistical association between PPD and the lactation (*P* = 0·212). The results showed statistically significant associations between PPD and being classified as a high-risk pregnancy (*P* = 0·003), in which 6·0 % of the cases mothers faced gestational hypertension, 3·3 % faced gestational diabetes and 2·0 % had pre-term labour. Previous use of oral or injection contraceptive methods did not indicate any significant differences between the two groups (*P* = 0·178).

**Table 1:** Comparison between cases and control

Profile	Cases (PPD) (%) = 150	Controls (non-PPD) (%) = 150	*P*-value
Housing			
Ownership	88·0	89·3	0·856
Rent	12·0	10·7	
Educational level of the mother			
Less than high school school	10·0	9·3	1·00
High school	52·7	52·7	
University and equivalent	37·3	38·0	
Occupational status of mother			
Employed	3·3	6·7	0·289
House wife	96·7	93·3	
Income category			
Deep poverty	95·3	92·0	0·388
Poor	3·3	4·0	
Not poor	1·3	4·0	
Sex of child			
Male	56·7	50·0	0·298
Female	43·3	50·0	
Worried about the sex of the baby	45·3	46·0	1·000
First child	32·0	25·3	0·250
Planned pregnancy	32·7	37·3	0·468
Type of delivery			
Normal	78·7	76·7	0·782
C.S.	21·3	23·3	
Lactation			
Breast milk	50·7	60·0	0·212
Formula feeding	4·0	2·0	
Mixed	45·3	38·0	
High-risk pregnancy (last pregnancy)	12·0	2·7	0·003
Contraceptive methods (oral or injection)	16·7	10·7	0·178
Undesired feeling in the last pregnancy	58·0	42·0	0·008
Taken psychological guidance services	2·0	20·7	<0·001
Mental health disorder in the last pregnancy	58·0	29·3	<0·001

Table 1 also shows that the mothers who had an undesired and uncomfortable feeling during the last pregnancy period were more susceptible to develop PPD after the baby delivery, as for 58·0 % of the mothers in the cases group were suffering from this such issues, and thus there were statistically significant differences between the two groups (*P* = 0·008). The relationship between the PPD and attending psychological guidance services was highly statistically significant (*P* < 0·001), since 20·7 % of the controls attended such services, while 98·0 % of the cases did not attend any psychological guidance services before or during the pregnancy. Moreover, there was a highly significant association between the PPD and having one of the mental health disorder in the last pregnancy (*P* < 0·001), 12·7 % of the mothers in the cases group were suffering from major depression during the last pregnancy, 22·0 % were suffering from eating disorders and 23·3 % were suffering from either anxiety, insomnia or panic.

Table 2 presents a highly significant differences between the two groups regarding the following CBC parameters that have a role in screening and diagnosis of the body iron status namely, Hb (*P* < 0·001), haematocrit (*P* < 0·001) and mean corpuscular volume (*P* = 0·013). The table shows that most of the participants were not having inflammation since 69·3 % of them had a normal hs-CRP level (<5 mg/l) and the rest of the participants had high hs-CRP level (>5 mg/l).

**Table 2:** Haematological and biochemical characteristics of cases and controls

Profile	Cases (PPD) *n* 150		Controls (non-PPD) *n* 150		95 % CI	*P*-value
	%/Mean	sd	(%)/Mean	sd		
Hb (g/dl)	11·47	1·22	12·08	1·03	−0·86, −0·35	< 0·001
HCT (%)	34·51	3·14	36·07	2·79	−2·23, −0·88	< 0·001
MCV (fl)	79·23	7·40	81·29	6·94	−3·69, −0·43	0·013
Ferritin	33·4	34·81	46·06	36·02	−20·71, −4·62	<0·001*
Low	43·30		15·30			<0·001
Normal	56·70		84·7			
hs-CRP	6·22	14·75	5·23	5·74	−1·55, 3·53	0·730*
Normal	69·30		69·30			1·00
High	30·7		30·7			
STfR	32·43	35·90	20·6	23·15	4·96, 18·69	<0·001*
Low	4·7		10·0			<0·001
Normal	60·0		75·3			
High	35·3		14·7			
sTfR/log ferritin index	7·29	6·44	10·46	4·99	0 356, 1·34	<0·001
ID	15·3		4·7			0·003
Non-ID	84·7		95·3			

HCT, haematocrit; MCV, mean corpuscular volume; sTfR, soluble transferrin receptors; ID, iron deficiency.

* Mann*–*Whitney test for non-parametric test.

The mean difference in the sTfR between the two groups was statistically significant and as shown in the table. The results show that cases have almost 12 units of sTfR higher than that of the controls. Moreover, the mean difference in the sTfR/log ferritin index was statistically significant and it was in favour of the control group. The sTfR/log ferritin index determined that most (84·7 %) of the mothers in the cases group were not categorised with ID and 23 mothers (15·3 %) were categorised with ID, while only 7 (4·7 %) mothers in the controls group were categorised with ID, and 95·3 % were not categorised with ID. Thus, there was a statistical significant differences in ID between the two groups (*P* = 0·003).

Based on the results of simple logistic regression (SLgR), independent variables with a *P*-value < 0·25 or any other important associated factors from previous studies were included in the multiple logistic regression (MLgR). Table 3 presents all the determinants appearing in the final model that remained significantly associated with the PPD status, namely, the psychological guidance services, the undesired feeling during the last pregnancy, the mental health disorder in the last pregnancy and the body iron status. The results of MLgR showed that the mothers who did not attend any psychological guidance services were 8·54 times more likely to develop PPD (OR^adj^ 8·54, (95 % CI 2·41, 30·21); *P* = 0·001), and that the mothers who had undesired feeling in the last pregnancy were 1·77 times more susceptible to suffer from PPD than the mothers who did not have that feeling (OR^adj^ 1·77, (95 % CI 1·04, 3·03); *P* = 0·034).

**Table 3:** Factors associated with postpartum depression from multiple logistic regression analysis (*n* 300)

Variables	Simple logistic regression			Multiple logistic regression				
	Odd ratio	95·% CI	*P*-value	Adjusted *β*	se	Adjusted OR	95 % CI	*P*-value
Absence of psychological guidance services	12·76	3·81, 42·78	<0·001	2·24	0·64	8·54	2·41, 30·21	0·001
Undesired feeling in the last pregnancy	1·91	1·21, 3·02	0·006	0·57	0·27	1·77	1·04, 3·03	0·034
Mental health disorder in the last pregnancy	–		< 0·001	–		–		0·001
Major depression	4·57	1·82, 11·47	0·001	1·27	0·49	3·56	1·36, 9·34	0·010
Eating disorders	3·97	1·97, 7·97	< 0·001	1·20	0·38	3·33	1·57, 7·06	0·002
Anxiety, insomnia or panic disorder	2·56	1·39, 4·72	0·003	0·93	0·34	2·54	1·3, 4·99	0·007
Body iron status	3·7	1·54, 8·91	0·004	1·18	0·48	3·25	1·26, 8·40	0·015

OR^adj^: OR adjusted.

The findings of the MLgR reveal that there was a statistically significant association between PPD and mental health disorders in the last pregnancy (*P* = 0·001), as the mothers who suffered from major depression or eating disorders were three times more likely to be PPD (OR^adj^ 3·56, (95 % CI 1·36, 9·34); *P* = 0·010) and (OR^adj^ 3·33, (95 % CI 1·57, 7·06); *P* = 0·002), respectively, while mothers who faced anxiety, insomnia or panic disorder were two times more likely to develop PPD than the mothers who did not suffer from mental health disorders (OR^adj^ 2·54, (95 % CI, 1·3, 4·99); *P* = 0·007). In investigating the association between PPD and body iron status, findings of this study imply that a significant association between PPD and body iron status existed (p = 0·015). The mothers who suffered from ID were three times more likely to have PPD (OR^adj^ 3·25, (95 % CI 1·26, 8·40); *P* = 0·015).

## Discussion

The recurrent wars on Gaza exacerbated the humanitarian crisis, unemployed in Palestine reached 31 %; of which 52 % in Gaza while the poverty percentage among Palestinian individuals according to consumption patterns was 29 %, of which 53 % of which was in Gaza, and almost three in four people are food-insecure^(27,28)^. As mentioned earlier, the degraded socio-economic situation and food-insecure and the political violence makes depression one of the top five causes of disability in the occupied Palestinian territory. The intended aims of the present study were to investigate the role of body iron status role in PPD, in addition to other factors.

The results of a prospective observational study by Chandrasekaran and his team showed that there was no difference in Hb or iron levels in women who had PPD compared to those without^(17)^. This contradicts the results of our study, since the results showed a highly significant association between PPD and Hb and haematocrit. In addition to a significant association with mean corpuscular volume (*P* = 0·013), which could be due to the small sample size in Chandrasekaran *et al.* study that included only 103 mothers. On the other hand, there were many studies that correspond to the results of our study regarding the significant association between PPD and Hb, as in the multivariate analysis of a study by Beard *et al.* which showed that the mother’s scores on the EPDS were significantly correlated with Hb and mean corpuscular volume^(29)^. In addition, another study that involved 352 Saudi postpartum mothers depends only on Hb testing, as its low level detect of anaemia among the delivered mothers. The results of the study suggested that early postpartum anaemia evident by low Hb level is a significant risk factor for PPD^(30)^.

In the current study, the ferritin level in mothers with PPD was lower than its level in mothers without PPD. Also, the results of this study showed a highly significant association between PPD and serum ferritin level of the postpartum mothers. The same significant differences were detected between mothers with PPD and mothers without PPD regarding sTfR and body iron status (sTfR/log ferritin index). The results of the study did not detect any significant association between PPD and the hs-CRP level (*P* = 1·00). This significant relationship can be explained by the biological role of iron in maintaining the brain and cognition health since iron is critical for adequate myelination, neurotransmitter metabolism and function, and neuronal cellular and oxidative processes^(17)^. Thus low iron level will lead to a deterioration of these functions and develop the clinical depression in mothers. Likewise, there were studies in which results are consistent with the results of our study as the Albacara *et al.* study that supports the role of iron in the aetiology of PPD. The study observed a strong association between ferritin and PPD^(14)^, while there was no significant association between CRP and PPD (*P* = 0·51). The association between PPD and iron was also detected in a randomised double-blind placebo-controlled trial by Sheikh *et al.* which concluded that providing early iron supplementation to the delivered mothers, who suffer from PPD, can significantly improve the iron stores and lead to significant improvement in PPD with a 42·8 % improvement rate during 6 weeks and that the persistence of PPD may be relevant to the lower postnatal ferritin levels in untreated mothers^(15)^. In contrast, results of study by Armony-Sivan *et al.* who investigated this association among Chinese mothers demonstrated that even in this large sample, no correlation reached statistical significance, as the correlations between the mothers iron biochemical tests (Hb, mean corpuscular volume, ferritin, sTfR and sTfR Index) during mid- or late-pregnancy or 3 d postpartum. EPDS scores shortly after delivery or at 6 weeks were also low. Besides that, EPDS scores in anaemic and non-anaemic mothers did not differ from the results of Armony-Sivan *et al.* study, which showed similarity in iron status in both groups (women with or without PPD)^(18)^. Thus, there was no relationship between the maternal iron status and PPD. Moreover, there was an exception for sTfR at mid- and late-pregnancy and EPDS at 6 weeks, as higher sTfR was related to lower depressive symptoms, which was opposite from what would be expected.

The current study found that the mothers who suffered from ID were three times more likely to have PPD. However, the discrepancy between our results and the results of the other studies regarding the biochemical part could be due to the differences in the methodologies of performing the test including the device and the kits that been used to assess the biochemical markers since some normal ranges could differ from kit to another. Furthermore, we could not find any study that depends on the sTfR/log ferritin index to assess body iron status and its association with PPD.

The study detected a highly significant association between PPD and having one of the mental health disorders in the last pregnancy including major depression, eating disorders, anxiety, insomnia or panic. Our results are consistent with previous studies, as in the retrospective controlled study by Morgan *et al.*, which showed that, during pregnancy, active bulimia (one of the eating disorders) is associated with postnatal depression, miscarriage and pre-term delivery. In addition, bulimia may be a treatable cause of the adverse obstetric outcome^(31)^. While in Stewart work that investigated the depression and anxiety showed that most PPD and anxiety were preceded by antenatal depression and anxiety. Furthermore, the antenatal anxiety also predicted PPD, even after controlling for antenatal depression^(32)^.

On the other hand, the results of our study showed a significant difference between PPD mothers and non-PPD mothers regarding the classification of the mother as high-risk pregnancy during the last pregnancy. This corresponds to the results of a study by Zadeh *et al.* which found that the prevalence of moderate-to-severe symptoms of depression and anxiety was higher among mothers who had a high-risk pregnancy^(33)^.

Overall, there was a discrepancy between the results of this study and the results of the numerous previous studies that investigated those factors. This could be due to many reasons as the variation in studies environment, habits, concepts and beliefs, in addition to the diversity regarding the methodology that has been used. Furthermore, the variation between the studies in selecting the cut-off point in the EPDS could be one of the most important reasons.

The results of the current study indicate that undesired feeling in the last pregnancy, mental health disorder and high-risk pregnancy are some of the PPD factors. These factors combined together can also worsen the condition of PPD in the affected mothers, as these factors are usually combined with affecting mother’s appetite for food. This will lead to a deficiency of some basic elements such as ID, which in turn, will aggravate the situation.

### Strengths

Choosing the mothers after a month of postpartum helped prevent overlapping with other postpartum psychiatric disorders including the postpartum blues and psychosis. Furthermore, the translated copy of the EPDS questionnaire including the used cut-off point (scoring of 10 and higher) was valid and pre-tested, whereas the framing of the questionnaire questions and their answers in addition to the selection of the cut-off point are considered critical in detecting the presence of PPD among the mothers.

Regarding the detection of ID, the effect of inflammation (high CRP concentration) on the ferritin was avoided, by the use of sTfR that reflects the functional iron compartment, and is not acute phase reactant. Moreover, the equation that was used (sTfR/log ferritin index) in detecting the body iron stores has been shown to be valid and superior to routine tests.

### Limitations

Despite all the precautions that were taken to make the participants feel safe and comfortable while answering the questions, it is believed that some of them were embarrassed to answer some questions correctly.

Using sTfR/log ferritin index as a proxy for iron status can be considered an addition point, but on the other hand, it can be considered a negative point, as it deprived the researchers of comparisons with other studies.

Case–control studies are an efficient method for the study of rare outcomes but suffer various limitations, including susceptibility to bias in recollection about exposure, and reverse causality

Due to the financial barriers, the biochemical tests were restricted to the selected tests. Other complementary tests could not be performed, as well as the anthropometric measurements could not be performed due to minimising interview times out of consideration for the circumstances of the participating mothers.

## Conclusion

The results showed that mothers who have ID are at high risk of developing PPD, as the mothers who suffered from ID were three times more likely to have PPD.

### Recommendations

The mothers and their families need to pay more attention to the mother’s food consumption before, during and after pregnancy. Primarily, ensure diversity in her consumed food and focus on foods rich in iron so she can meet her recommended daily intake. Furthermore, the mother must be aware of her feelings and psychological state and should not hesitate to attend the available psychological guidance services before, during or after the postpartum period. Routine physical and psychological check-ups during pregnancy period are highly recommended particularly for mental health disorders.
